# Author Correction: Direct evidence for microbial-derived soil organic matter formation and its ecophysiological controls

**DOI:** 10.1038/s41467-018-06427-3

**Published:** 2018-09-24

**Authors:** Cynthia M. Kallenbach, Serita D. Frey, A. Stuart Grandy

**Affiliations:** 1 Department of Natural Resources and the Environment, University of New Hampshire, Durham, New Hampshire 03824 USA; 2 Soil and Crop Sciences Department, Colorado State University, Fort Collins, Colorado 80523 USA

Correction to: *Nature Communications*; 10.1038/ncomms13630; published online 28 November 2016

In the originally published version of this Article, financial support was not fully acknowledged. The PDF and HTML versions of the Article have now been corrected to include support from the NSF Long-term Ecological Research Program (DEB 1637653) at the Kellogg Biological Station and from Michigan State University AgBioResearch.

