# Supplementary material for: Direct evidence for microbial-derived soil organic matter formation and its ecophysiological controls
Source: Nat Commun. 2016 Nov 28;7:13630. doi: 10.1038/ncomms13630 (PMC5133697; doi:10.1038/ncomms13630)
Supplement: Supplementary Information — Supplementary Figures 1-4, Supplementary Tables 1-4 and Supplementary Notes 1-2 [file ncomms13630-s1.pdf]

Supplemental Information

Supplemental Figure 1| Soil microbial biomass, enzymes and respiration. Mean microbial biomass (MBC) (a), microbial CO_2 respiration rates (b), and potential β-glucosidase (BG) (c) and phenol oxidase enzyme activities (d) across substrate and mineralogy treatments at 6, 9, 12, and 15 mo. Substrate (sugar, syringol or DOC) prefixes ‘K’ and ‘M’ are kaolinite and montmorillonite. Error bars are one standard error (Experimental replication *n* = 5).

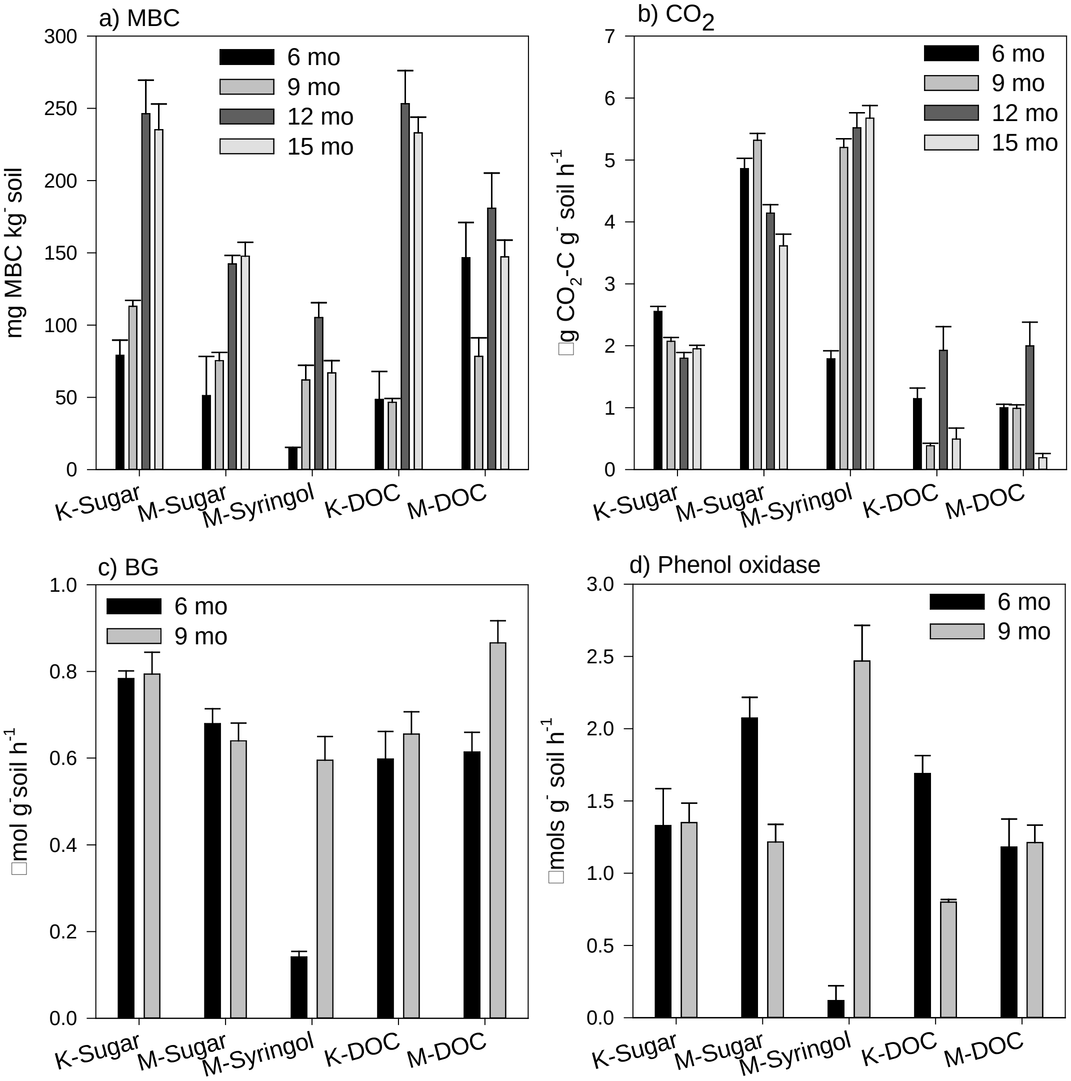

Supplemental Figure 2| Percentage of novel organic compounds. The percent of identified compounds that are unique to inoculated soils (not present in unprocessed substrates) relative to the total number of identified compounds. The percent of unique compounds are categorized by compound class for kaolinite (a) and montmorillonite (b). The total percent unique compounds were significant within clay treatments (ANOVA: $P < 0.05$). The (*) indicates significant differences ($P < 0.05$) between sugar and dissolved organic carbon (DOC) or syringol treated soils within clay treatments and (†) indicates significant differences between syringol and DOC treated soils.

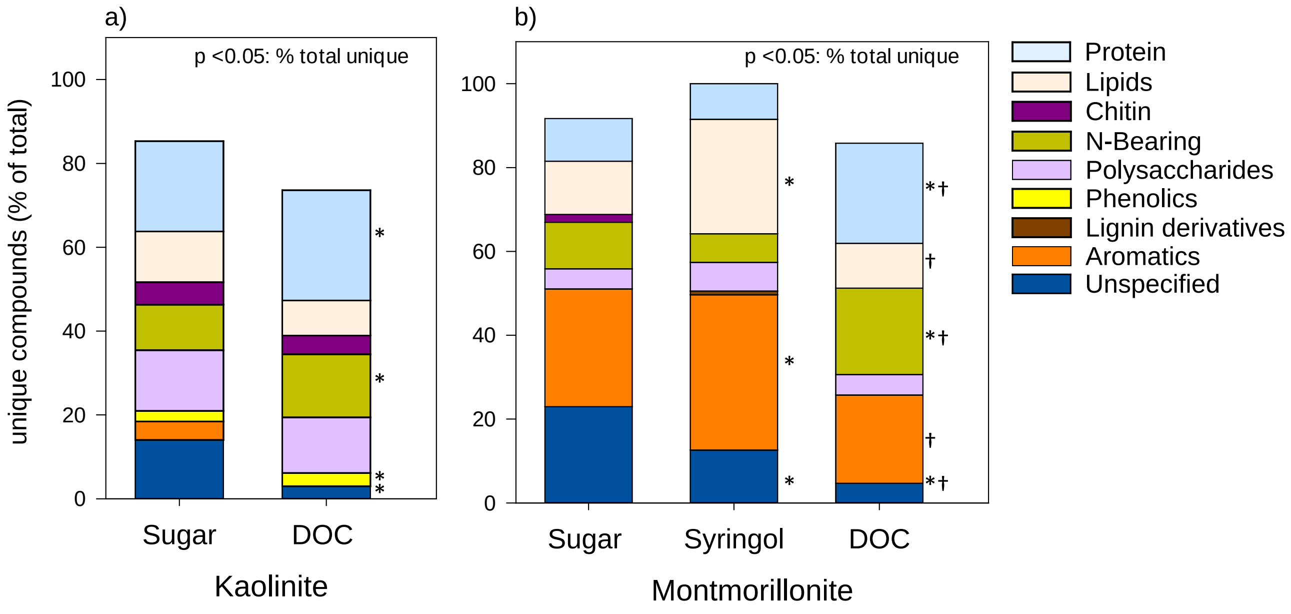

Supplemental Figure 3| Soil microbial community composition. Non-metric multidimensional scaling (NMDS) ordination of phospholipid fatty acid (PLFA) biomarkers for substrate and clay treatments at 12 and 15 mo. Open symbols are kaolinite and closed symbols are montmorillonite soils (Stress = 9, Monte Carlo *P* <0.05; MRPP *P* <0.0001). Pearson's r = -0.97 for bacterial relative abundance and 0.96 for fungal relative abundance.

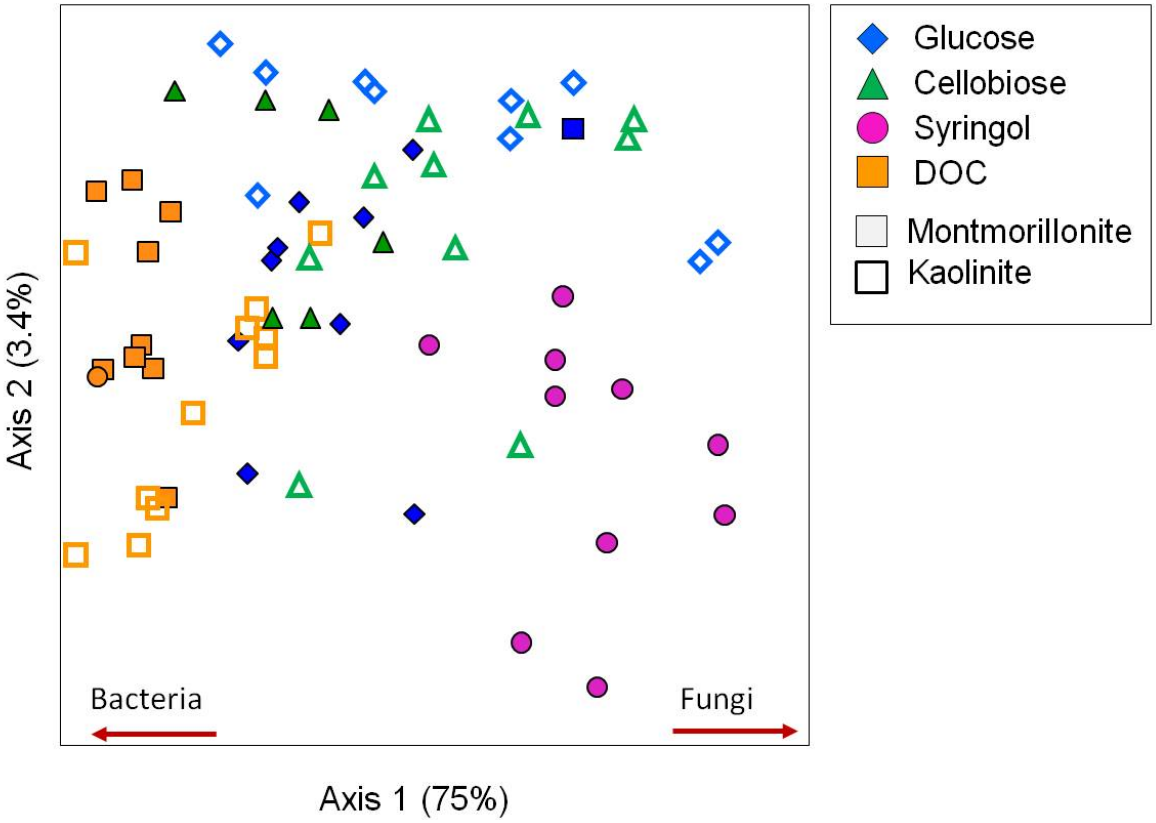

Supplemental Figure 4| Relationships between soil organic matter chemistry, soil carbon concentrations, microbial physiology and fungi. Pearson correlations for the sugar-treated soils: soil C (SOC) (%) and PLFA fungal:bacterial ratios at 12 and 15 mo. (a) SOC and microbial C use efficiency at 15 mo. (b); SOC and lipid (c) and protein (d) relative abundances at 18 mo.; lipid and fungal relative abundance (e) and microbial carbon use efficiency (f) at 15 mo.

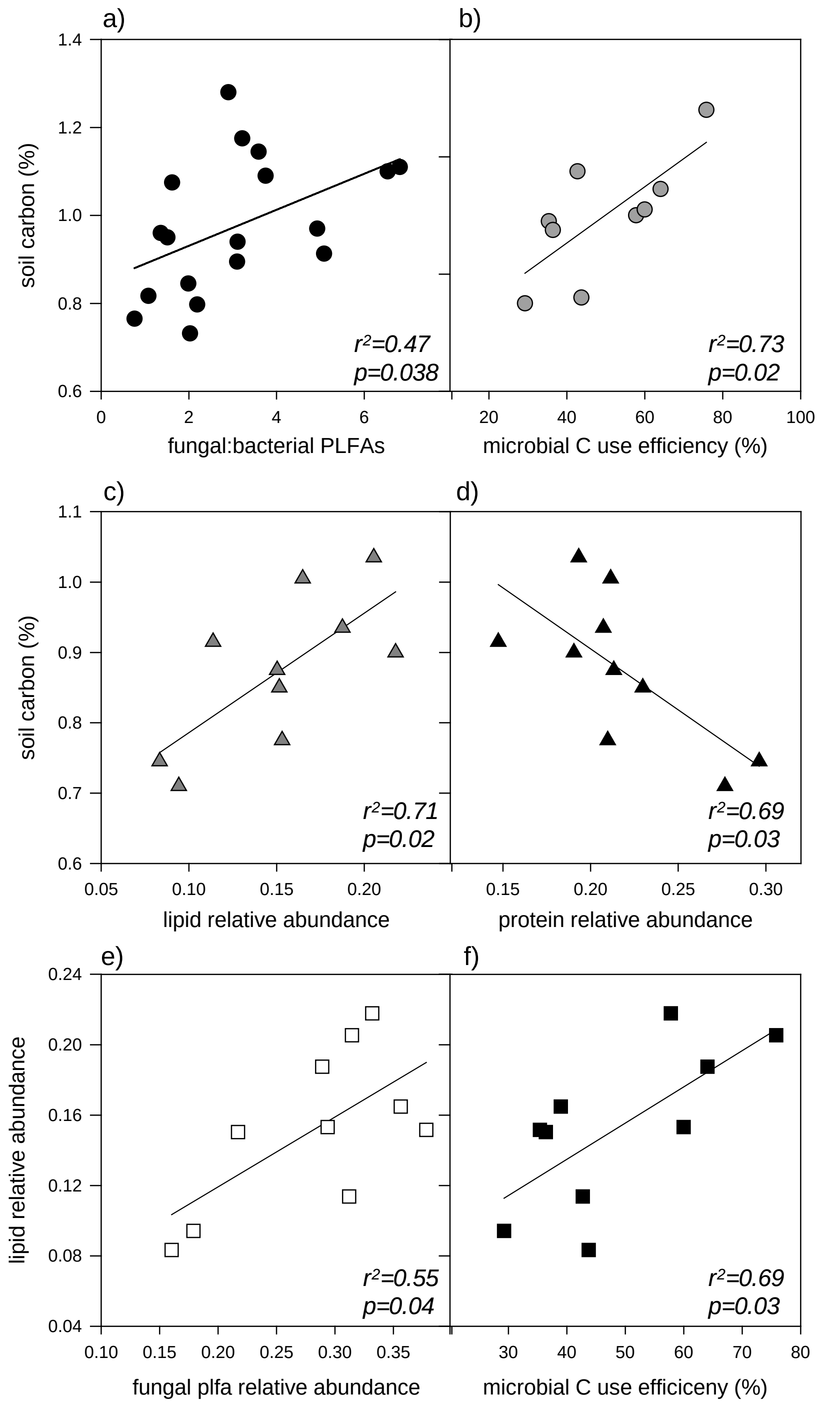

Supplemental Table 1| Relative abundances of soil organic matter compound classes. The relative abundances (%) for each compound class in the pure substrates and the model soils. Differences between clay type are indicated by different capital letters and differences in soils between substrate input within a model soil clay type are indicated by lower case letters (ANOVA: P <0.05). Values are treatment mean (n = 5). These data correspond to Figure 1 in the main article.

Substrate/Soil	Polysacch.	Protein	Chitin	Lipids	N-Bearing	Phenolics	Lignin	Aromatics	Unspec.
	relative abundances (%)								
Sugar	84.06	0.00	1.66	1.57	2.95	0.54	0.62	0.45	8.15
Kaol-sugar	23.85^{Ba}	22.54^{Ba}	5.19^{Bb}	14.61^{Aa}	10.2^{Aa}	1.32^{Aa}	0.26^{Aa}	6.29^{Ab}	15.75^{Aa}
Mont-sugar	12.23^{Aa}	13.31^{Ab}	0.03^{Aa}	13.80^{Aa}	8.14^{Aa}	1.64^{Aa}	0.45^{Ba}	28.08^{Bb}	22.32^{Ba}
Syringol	0.52	0.00	0.00	0.05	0.00	0.00	99.26	0.00	0.17
Mont-syringol	8.81^{a}	8.06^{a}	0.00^{a}	23.35^{a}	7.15^{a}	0.00^{a}	1.00^{b}	25.84^{b}	25.79^{b}
DOC	24.00	2.54	0.64	23.10	1.71	1.05	10.80	2.44	33.72
Kaol-DOC	17.54^{Aa}	26.47^{Bb}	2.70^{Ba}	20.31^{Bb}	10.47^{Aa}	1.3^{Ba}	2.58^{Bb}	3.96^{Aa}	14.67^{Aa}
Mont-DOC	13.72^{Aa}	20.48^{Ac}	0.00^{Aa}	12.76^{Aa}	17.31^{Bb}	0.00^{Aa}	1.07^{Ab}	15.2^{Ba}	19.46^{Ba}

Supplemental Table 2| The most abundant compounds in substrates and model soils. The top three of the most abundant compounds (relative abundance of total-%) within the aromatic, unspecified, N-bearing, and polysaccharide classes for natural soil fungal biomass [†], pure sugar and syringol substrates and model soils receiving sugar or syringol. Total abundances for the classes are shown in the gray rows. Values are treatment mean (*n* = 5).

Aromatics		Unspecified		N-bearing		Polysaccharides	
compound	(%)	compound	(%)	compound	(%)	compound	(%)
Fungal biomass†	2.6		16.6		5.7		36.9
m-xylene	0.90	Toluene	6.81	Aniline	2.80	Furfural	10.70
Oxirane, ethenyl-	0.70	Phosphonic acid	2.92	4-Pyridine carboxaldehyde	0.75	Furfural, 5-methyl-	8.90
Acetophenone	0.27	1,3,5,7-Cyclooctatetraene	2.89	3-Pyridinol	0.62	4H-Pyran-4-one, 3-hydroxy-2-me	2.71
Sugar	0.45		8.2		3.0		84.1
Benzofuran	0.13	Hydroquinone	3.50	N-Butyl-tert-butylamine	1.85	Furfural	39.75
Acetophenone	0.11	Pyruvaldehyde	2.97	2,5-Furandione, 3-me	0.42	Levoglucosan	14.93
Benzaldehyde	0.10	Phosphonic acid	0.71	1,4-Benzenediamine	0.36	2(5H)-Furanone	8.85
Montmorillonite Sugar	28.1		22.3		8.1		11.3
Benzene, 1,2,3-trimethyl-	6.80	Toluene	8.86	Propane, 2-nitro-	2.27	Furfural	3.84
m-xylene	5.64	2,3,6-Trimethylnaphthalene	2.45	Aniline	1.42	Butanal, 2-methyl-	1.53
Benzene, 1,2,3,4-tetramethyl-	2.38	Indane	2.06	Hexanedinitrile	1.23	Furan, 2,5-dimethyl-	1.02
Kaolinte Sugar	6.3		15.8		10.2		23.8
Benzene, butyl-	1.67	Toluene	5.45	1H-Pyrrole, 3-me	1.92	Furfural	4.24
Benzene	0.80	Phosphonic acid	3.71	Pyridine 3-me	1.84	2-Cyclopenten-1-one, 2-hydroxy-3-me	2.36
Phenol, 3,4-dimethyl-	0.67	1,3,5,7-Cyclooctatetraene	2.05	Aniline	1.78	Furfural, 5-methyl-	2.08
Syringol	0.00		0.17		0.00		0.53
		1,3,5,7-Cyclooctatetraene	0.03			Levoglucosan	0.48
		Mequinol	0.01			Furfural	0.03
		1-Undecanol	0.00			2-Cyclopenten-1-one, 2-hydroxy-3-me	0.02
Montmorillonite Syringol	25.8		25.8		7.2		8.8
Benzene	6.72	Toluene	20.44	Propane, 2-nitro-	5.82	Butanal, 2-methyl-	6.19
m-xylene	5.34	1,3,5,7-Cyclooctatetraene	4.87	Acetonitrile	0.90	Furan, 2,5-dimethyl-	2.07
Benzene, 1,2,3-trimethyl-	4.44	2,3,6-Trimethylnaphthalene	0.94	5- Dimethylaminopyrimidine	0.56	Cyclopentanone	0.29

[†]The fungal biomass was derived from a basidiomycete culture isolate from soils collected at the Long Term Experimental Research site at Harvard Forest (Petersham, MA USA).

Supplemental Table 3| Two- and one-way ANOVA results for soil carbon, microbial community and SOM chemistry.

	Two-way ANOVA						One-way ANOVA			
	Main Effects				Interaction		Main Effect: Substrate			
	Clay		Substrate		Clay x Substrate		Montmorillonite		Kaolinite	
**Variables**	F-value	*P*	F-value	*P*	F-value	*P*	F-value	*P*	F-value	*P*
**Soil Carbon**										
Final SOC (18 mo.)	0.02	0.89	26.61	**<0.001**	3.48	**0.05**	26.96	**<0.001**	5.65	**0.02**
Chemical SOC stability	1.46	0.24	1.40	0.27	1.32	0.29	3.71	**0.04**	0.01	0.99
Biological SOC stability	11.45	**0.00**	5.64	**0.01**	5.24	**0.01**	3.99	**0.03**	0.08	0.93
**Microbial Community**										
Fungal relative abundance	20.44	**<0.001**	15.90	**<0.001**	6.12	**0.01**	46.42	**<0.001**	7.64	**0.01**
Gram positive relative abundance	5.39	**0.03**	2.21	0.13	1.87	0.18	11.03	**<0.001**	2.75	0.10
Gram negative relative abundance	20.64	**<0.001**	14.50	**<0.001**	9.72	**<0.001**	35.73	**<0.001**	6.03	**0.02**
CUE at 9 months	3.12	0.09	2.74	0.09	9.79	**<0.001**	18.22	**<0.001**	9.05	**0.004**
CUE at 15 months	1.16	0.29	1.49	0.25	6.66	**0.01**	10.98	**<0.001**	1.31	0.31
**SOM Chemistry**										
Lipid relative abundance	2.04	0.17	1.14	0.34	1.12	0.35	1.33	0.31	2.40	0.14
Protein relative abundance	64.74	**<0.001**	14.62	**<0.001**	2.55	0.10	25.00	**<0.001**	2.49	0.13
Polysaccharides relative abundance	12.94	**0.00**	0.42	0.66	1.34	0.28	0.27	0.85	5.80	**0.02**
N-bearing relative abundance	0.76	0.39	9.89	**<0.001**	8.37	**0.002**	8.67	**0.002**	0.64	0.55
Aromatics relative abundance	235.42	**<0.001**	20.51	**<0.001**	9.76	**0.001**	8.79	**0.002**	4.23	**0.04**
Chitin relative abundance	140.09	**<0.001**	5.76	**0.01**	5.60	**0.01**	1.00	0.42	6.26	**0.02**

Supplemental Table 4| Soil cumulative respiration. Total CO_2-C respiration for 3, 6, 9, 12, and 15 months for glucose, cellobiose, syringol, or DOC-treated kaolinite and montmorillonite soil and the total amount of substrate-C added over a 15-month period. Values are treatment mean (Experimental replication *n* = 5) with standard deviation in parentheses.

		Cumulative Respiration†			
	Month	Glucose	Cellobiose	Syringol	DOC
Kaolinite		mg CO_2-C g^{-1} soil			
	3	3.18 (±0.32)	3.42 (±0.36)	.	1.98 (±0.23)
	6	8.53 (±0.64)	9.56 (±0.98)	.	2.90 (±0.21)
	9	13.49 (±0.79)	14.12 (±1.13)	.	4.10 (±0.22)
	12	20.66 (±0.62)	20.55 (±1.28)	.	7.85 (±0.29)
	15	24.80 (±0.71)	24.41 (±1.22)	.	8.69 (±0.35)
Montmorillonite					
	3	4.27 (±0.24)	4.75 (±0.18)	1.12 (±0.25)	1.30 (±0.02)
	6	18.40 (±0.55)	19.35 (±0.30)	8.61 (±1.11)	3.05 (±0.03)
	9	30.05 (±0.98)	31.80 (±0.33)	21.95 (±1.46)	5.61 (±0.22)
	12	37.88 (±1.2)	40.56 (±0.66)	28.84 (±1.60)	10.10 (±0.33)
	15	44.12 (±1.68)	42.91 (±1.5)	34.92 (±2.45)	10.86 (±0.32)
Total substrate-C input at 15 mo.				46.9	

†Cumulative values are based on weekly and bi-weekly short-term measurements collected primarily to monitor microbial activity. Carbon mass balances are reported in Table 1 of the primary text.

Supplemental Note 1| SOM molecular chemistry

Some py-GC/MS compounds originate from multiple classes and these are designated as *unspecified origin*, which makes up 20-28% of the sugar- and syringol-treated samples after 18 mo. Unspecified compounds are also detected in the unprocessed substrates. In the sugar substrates these unspecified compounds are presumably pyrolysis products of polysaccharides. However, in the incubated soils (at 18 mo.) several of the most abundant unspecified compounds are likely pyrolysis products of microbes rather than sugar substrates and were not present in the unprocessed substrates or when substrates were combined with uninoculated clay mixtures (Supplemental Table 2).

In the unprocessed glucose and cellobiose substrates, three polysaccharide compounds (furfural, levoglucosan, and 2(5H)-furanone) accounted for 66% of total identified compounds. The remaining 51 identified substrate compounds individually contributed less than 5%. These three dominant compounds in the pure sugar substrates only constituted 8 and 4% in the kaolinite and montmorillonite sugar-treated soils after 18 mo. Additionally, we analyzed the chemistry of natural fungal biomass to represent a general microbial end-member and found that furfural represents 11% abundance of the fungal biomass compounds (Supplemental Table 2). We also observed the three most abundant unspecified compounds (toluene, phosphonic acid, and 1,3,5,7-cyclooctatetraene) were the same for both the fungal biomass and all model soils. In syringol soils toluene and 1,3,5,7-cyclooctatetraene, alone made up a mean 25% relative abundances across all compound classes but were absent in the pure syringol (Supplemental Table 2).

Supplemental Note 2| Microbial community physiological metrics

Based on the replacement of syringol substrate with microbial residues and novel compounds and the high microbial activity rates, it appears that the microbial community in the syringol treated soils is well-adapted to utilizing syringol-C. However, our CUE estimates are based on glutamic acid uptake rather than the substrate that the community was exposed to. Unlike in the glucose and cellobiose treated soils, the benzene ring in the syringol will first need to be enzymatically cleaved before microbial uptake. In this study we were more interested in C allocation towards new biomass growth rather than total substrate use efficiency, which would necessarily include the allocation of the substrate to all aspects of microbial use (i.e., enzyme, biofilms and metabolite production). By using a substrate that can be directly taken up by microbes, we are able to minimize the amount of C allocation to enzyme production to better target the efficiency of the community to use a substrate for new biomass synthesis. Thus, in the syringol treatment, some C is certainly allocated towards enzyme production but the ^{13}C-glutamic acid results suggest that, once cleaved, the community can more efficiently synthesize new biomass from incorporated C.
